# Exploring SLC16A1 as an Oncogenic Regulator and Therapeutic Target in Cholangiocarcinoma

**DOI:** 10.7150/jca.95258

**Published:** 2024-05-20

**Authors:** Jianxin Huang, Fahui Liu, Donghua Liu, Shihang Tang, Dongyan Shen

**Affiliations:** 1 Department of Clinical Laboratory, Fujian Provincial Hospital, Shengli Clinical Medical College of Fujian Medical University, Fuzhou, 350001, China.; 2 Xiamen Cell Therapy Research Center, The First Affiliated Hospital of Xiamen University, School of Medicine, Xiamen University, Xiamen 361003, Fujian Province, China.

**Keywords:** Cholangiocarcinoma, SLC16A1, 5-Fluorouracil, Bioinformatics Analysis

## Abstract

Cholangiocarcinoma (CCA) is a primary malignant tumor of the liver, typically diagnosed in advanced stages. Surgical resection remains the principal treatment method in clinical practice. Regrettably, the majority of patients receive their diagnosis at an advanced stage, making surgical intervention unfeasible. While chemotherapy serves as the main palliative treatment for advanced CCA, its effectiveness is significantly limited due to the rapid development of chemoresistance. Studying the pathogenesis of CCA and new resistance targets is crucial for improving clinical outcomes. In our current study, we first identified the expression of SLC16A1 in the transcriptome and proteome of human tumors and found abnormal expression of SLC16A1 in various human cancers. Subsequently, we focused our attention on the role of SLC16A1 in CCA. Utilizing bioinformatics analysis, we pioneered the identification of the clinical significance of SLC16A1 in this type of cancer. Specifically, higher expression levels of SLC16A1 were observed in CCA patients with venous invasion and higher T and M stages. Additionally, patients with higher SLC16A1 expression had poorer prognoses. These results suggest the oncogenic role of SLC16A1 in CCA. Further immune infiltration analysis revealed a significant correlation between SLC16A1 and the infiltration levels of cells like neutrophils and macrophages in the tumor microenvironment, indicating SLC16A1's potential involvement in regulating the tumor immune microenvironment of CCA. Moreover, results from functional and pathway enrichment analyses revealed that SLC16A1 might affect clinical outcomes in CCA patients by participating in drug metabolism processes. Finally, through further *in vitro* and *in vivo* experiments, we confirmed that SLC16A1, as an oncogene in CCA, promotes the growth of CCA cells and chemoresistance. Knocking down SLC16A1 inhibited the growth of CCA cells and enhanced their sensitivity to 5-Fluorouracil (5-FU). Overall, this study reveals the key role of SLC16A1 in the development of CCA and highlights its significance as a potential target for improving treatment efficacy and chemotherapy sensitivity.

## Introduction

Cholangiocarcinoma (CCA), originating from the bile duct tree, ranks as the second most common primary malignant liver tumor, following hepatocellular carcinoma 1. Globally, the incidence of CCA exhibits significant geographical variations, likely tied to a complex interplay of genetic factors, environmental influences, and lifestyle choices. Currently, surgical resection is the only treatment considered effective 2. Regrettably, the majority of patients are diagnosed in the advanced stages, making surgery infeasible. Although chemotherapy is a common treatment modality for CCA, it primarily serves a palliative role, only modestly extending patient survival and often accompanied by the issue of chemoresistance 3. Therefore, a deeper understanding of the pathogenesis of CCA and the exploration of new resistance targets are crucial for developing more effective treatment strategies and improving the prognosis of CCA patients.

SLC16A1, also known as Monocarboxylate Transporter 1 (MCT1), is a gene located within the human genome 4. The protein encoded by this gene is a member of the monocarboxylate transporter family, which primarily functions in transporting small molecules like lactate and ketone bodies across cell membranes 5. These molecules play a significant role in numerous physiological processes, including energy metabolism and cell signaling 6. The unique function of SLC16A1 lies in its role in the transport of lactate. Lactate is produced by muscle cells under anaerobic conditions, such as during intense exercise, and is reconverted into energy upon the restoration of oxygen supply. Under normal circumstances, SLC16A1 facilitates the transfer of lactate from the cells where it is produced to those where it is needed, such as cardiac and hepatic cells. However, the role and function of SLC16A1 in human tumors may undergo significant alterations. Cancer cells often rely on the process known as 'aerobic glycolysis' or the 'Warburg effect' to produce energy, even under conditions of ample oxygen 7. This process generates a high amount of lactate, which must be expelled from cancer cells through SLC16A1 to avoid cytotoxicity 8. Moreover, SLC16A1 plays a role in tumor metastasis. For instance, SLC16A1 can interact directly or indirectly with components of the NF-kB signaling pathway, facilitating the survival and metastatic activities of tumor cells 9. Additionally, blocking the lactate influx mediated by SLC16A1 in endothelial and osteoclast cells impairs tumor-induced angiogenesis and bone resorption, thereby inhibiting tumor progression 10. Studies have observed that inhibiting or knocking down SLC16A1 in mixed cancer cell-fibroblast xenografts in mice can delay tumor growth 11. In summary, SLC16A1 has been reported to play a pivotal role in the development and progression of a considerable number of human cancers, making it an important subject of cancer research and a potential therapeutic target.

Despite extensive research demonstrating the critical role of SLC16A1 as an oncogene in various cancers, its role in the progression and treatment of CCA remains unclear. In this study, we utilized bioinformatics tools and functional experimental methods to preliminarily unveil the significant role of SLC16A1 as an oncogene in CCA. Our findings also suggest that SLC16A1 could serve as a potential therapeutic target, potentially enhancing the sensitivity of CCA patients to chemotherapy and reducing the occurrence of drug resistance. This research paves new pathways in the treatment study of CCA, offering crucial scientific evidence for future clinical applications.

## Materials and Methods

### Bioinformatics Analysis

In this study, we obtained TPM format RNAseq data from TCGA and GTEx projects via the Toil process from the UCSC XENA database (https://xenabrowser.net/datapages/). This data was utilized to compare the differential expression of SLC16A1 in human cancer tissues and corresponding normal tissues. Our specific approach involved extracting TCGA data under the pan-cancer category and corresponding normal tissue data from GTEx. All data were subjected to log2(value+1) transformation before analysis. The inclusion criteria for patients from TCGA-CHOL were as follows: The primary lesion had to be CCA, and patients with incomplete follow-up information were excluded.

To analyze the protein expression levels of SLC16A1 in normal and tumor samples across different cancer types, we employed data from the Clinical Proteomic Tumor Analysis Consortium (CPTAC) (https://proteomics.cancer.gov/programs/cptac). Additionally, the Human Protein Atlas (HPA) database (https://www.proteinatlas.org/) 12 was used to investigate the expression of SLC16A1 in various immune cells. Single-cell RNA sequencing data (GSE12345) from the TISCH database 13 (http://tisch.comp-genomics.org/home/) were used to reveal the expression characteristics of SLC16A1 at the single-cell level. Furthermore, to explore the clinical significance of SLC16A1 in CCA, we employed the BEST online analysis platform 14 (https://rookieutopia.com/app_direct/BEST/) for processing all related clinical data and their visualization results. The full name of each cancer type and abbreviations are provided in Table S1.

In the study of SLC16A1's function and its association with immune cells, our primary focus was on the TCGA-CHOL cohort. We extracted RNAseq data and corresponding clinical information for 35 CCA samples from The Cancer Genome Atlas (TCGA) dataset (https://portal.gdc.com). The CCA samples were divided into high and low-expression groups based on the median expression level of SLC16A1. The computation of differentially expressed genes was accomplished using the Limma package in R software. A threshold of "P value < 0.05 and log2(fold change) > 1 or log2(fold change) < -1" was set for selecting differentially expressed genes. For the functional enrichment analysis of these genes, we utilized the GO and KEGG databases to analyze the differentially expressed genes. We aimed to explore the signaling pathways and functional roles potentially involving SLC16A1. Subsequently, based on the ssGSEA algorithm, we calculated the level of immune cell infiltration in the TCGA-CHOL cohort using 24 types of immune cell markers provided in the literature. Additionally, we employed the IMPACT online database to analyze differences in immune-related and cancer-related signaling pathway activities between the high and low SLC16A1 expression groups 15.

Finally, for the analysis of drug sensitivity, we gathered data on the IC50 values of 265 small molecule drugs for 860 cell lines, along with mRNA gene expression data for SLC16A1, SLC16A2, SLC16A3, and SLC16A4 from the Genomics of Drug Sensitivity in Cancer (GDSC) database. By integrating the mRNA expression data with drug sensitivity data, we examined the correlation between gene expression levels and drug IC50 values. All relevant visualization analyses were conducted using the "Drug" module of the GSCA database 16. This approach allowed us to establish a connection between gene expression profiles and the responsiveness of various cancers to specific chemotherapy agents, thereby contributing to a more tailored and effective approach to cancer treatment strategies.

### Plasmids and Reagents

The primary antibodies used in this study were sourced from Proteintech (20139-1-AP) for SLC16A1, Santa Cruz Biotechnology (sc-23900) for Ki-67, and Cell Signaling Technology (#9662) for Caspase-3. The secondary antibody was obtained from Millipore (Billerica, MA, USA). The plasmids shSLC16A1-1, shSLC16A1-2, and the control plasmid shCtrl were acquired from Miaoling Biology (Wuhan, China). The chemotherapeutic agent 5-Fluorouracil (5-FU) was procured from Macklin (Shanghai, China). The Annexin V-FITC/PI Apoptosis Detection Kit was purchased from BD Biosciences (San Diego, CA, USA). The CCK-8 assay kit was obtained from MCE (USA). Crystal violet staining solution and the IHC kit were sourced from MXB (Fuzhou, China). The MiniPrep DNA Extraction Kit was bought from TIANGEN (Beijing, China). Lipofectamine 3000 was purchased from Thermo Fisher Scientific (USA).

### Cell Culture

QBC939 cells were provided by Professor Shuguang Wang from the Southwest Hospital of the Third Military Medical University. The HuCCT1 cell lines were kindly donated by Chun-Dong Yu's laboratory at Xiamen University. RPMI1640, Trypsin-EDTA, Penicillin, and Streptomycin were all purchased from Gibco (Carlsbad, CA, USA). Fetal Bovine Serum (FBS) was obtained from ExCell Bio (Shanghai, China). Both QBC939 and HuCCT1 cell lines were cultured in RPMI1640 medium, while the remaining cell lines were maintained in high-glucose DMEM. Both media were supplemented with 10% FBS and 1% antibiotics (100 u/ml Penicillin and 100 μg/ml Streptomycin). All cell lines were incubated in a cell culture incubator at 37°C and 5% CO_2_.

### Plasmid Amplification and Cell Transfection

For plasmid amplification, 1 μg of each plasmid (shSLC16A1-1, shSLC16A1-2, and the control plasmid shCtrl) was co-incubated with 10 μL of competent DH5α cells. This allowed for the integration of plasmid DNA into the competent cell DNA. Single clone strains capable of growth were selected using ampicillin and subsequently expanded in culture. Bacterial cells were lysed, and their DNA was extracted to obtain the plasmids shSLC16A1-1, shSLC16A1-2, and shCtrl. After verifying the sequences, these plasmids were used to transfect target cells. QBC939 and HuCCT1 cells were transfected with these plasmids using Lipofectamine 3000. The successfully transfected cells were then used for subsequent experimental studies.

### Drug Treatment

The 5-Fluorouracil (5-FU) solution was dissolved in PBS and sonicated to ensure complete dissolution, creating a 20 mM stock solution of 5-FU. This stock was then diluted to various final concentrations in the culture medium for use. All cells, after 24 hours of adherence, were treated with 5-FU, followed by subsequent assessments 24 hours post-treatment.

### Immunohistochemical Staining (IHC)

Samples including 3 cases of cholangiocarcinoma, 1 cases of adjacent non-tumor tissue, 1 case of cholecystitis, and 1 case of normal bile duct tissue were collected from The First Affiliated Hospital of Xiamen University. The criteria for the selection of these specimens are as follow: (1) Each patient was treated for the first time, with no prior exposure to radiotherapy, chemotherapy, or targeted anticancer drugs before their surgeries. (2) The diagnosis for all these cases was cholangiocarcinoma, confirmed not only by the primary lesion identification but also through the pathological assessment. The study was conducted in accordance with the guidelines of the Declaration of Helsinki and was approved by the Ethics Committee of The First Affiliated Hospital of Xiamen University (Approval number: XMYY-2022KYSB003).

Fresh clinical specimens are processed through fixation, dehydration, embedding, and sectioning. Subsequently, the sections are dewaxed, rehydrated, subjected to antigen retrieval, and treated to inactivate endogenous peroxidases. Following the instructions provided with the antibodies, the tissue sections are incubated overnight at 4°C with primary antibodies (1:200). Then, in accordance with the IHC staining protocol, they are incubated with secondary antibodies at 37°C for 30 minutes. Finally, the sections are stained with DAB chromogen and counterstained with hematoxylin to visualize the cell nuclei. After staining, the tissue sections undergo dehydration, are mounted, and photographed for preservation. In addition, the same IHC staining protocol was applied to the paraffin-embedded tissue sections from mouse tumors. The primary antibodies were diluted as follows: Ki-67 (1:50) and Caspase-3 (1:500).

### Clonogenic Assay

For the clonogenic assay, 1×10^3^ cells per well were seeded in six-well plates. The plates were incubated for 7 days to allow for colony formation. After the incubation period, cells were washed with PBS and fixed with methanol for 10 minutes. Post-fixation, the cells were washed again with PBS and stained with 0.1% crystal violet at room temperature for 15 minutes. Excess stain was washed off, and the plates were left to dry. Finally, cell colonies were counted and photographed under a microscope.

### Xenograft Transplantation

Nude mice (BALB/c, 14-16g) were acquired from SLAC Laboratory Animal Co. Ltd. (Shanghai, China). All experimental procedures were conducted in accordance with the approved animal handling protocols of the Laboratory Animal Center of Xiamen University and received approval from the Animal Ethics Committee of Xiamen University [XMYY-2022KYSB003]. The mice were randomly assigned into two groups. Each mouse was subcutaneously injected with either 6 × 10^6^ QBC939 shCtrl cells or QBC939 shSLC16A1 cells. The tumor volume and body weight of the mice were measured daily. Tumor volume was calculated using the formula: Volume = Length × Width^2 × 0.52. On day 21, the mice were euthanized, and the tumors were excised and photographed.

### Cell Viability Analysis

For this analysis, 5×10^3^ cells per well were seeded into a 96-well plate. These cells were then treated with various concentrations of 5-FU for 24 hours. Following this treatment, CCK-8 solution was added to each well according to the manufacturer's instructions. After incubating at 37°C for 4 hours, the absorbance at 450 nm was measured using a microplate reader to determine cell viability.

### Flow Cytometry Analysis of Cell Apoptosis

The detection of apoptotic cells was conducted according to the instructions of the FITC-Annexin V/PI staining kit. Initially, 5×10^4^ cells per well were seeded in a six-well plate and cultured for 24 hours. Different concentrations of 5-FU were then added to the medium, and the cells were further incubated for 24 hours. All cells were collected and centrifuged at 1000 rpm for 5 minutes, followed by two washes with PBS. The cells were then resuspended in 100 μL of Binding Buffer. Subsequently, 10 μL of FITC-Annexin V and PI dye were added to the suspension. The cells were incubated in the dark at room temperature for 15 minutes, after which an additional 50-100 μL of Binding Buffer was added. Finally, the cells were analyzed using a flow cytometer.

### Statistical Analysis

In addition to the statistical methods already mentioned, the rest of the experimental results are presented as mean ± SD. Survival analysis is performed using the log-rank test. Differences between two groups of data are compared using a two-tailed Student's t-test or Wilcoxon rank-sum test. For comparisons involving multiple groups, analysis of variance (ANOVA) is used. All statistical analyses are conducted using R or GraphPad Prism software, with a P-value < 0.05 considered statistically significant.

## Results

### Clinical features of the patients from TCGA-CHOL

A total of 35 CCA patients with clinical information and gene expression data were acquired from TCGA-CHOL database. The detailed clinical information includes Pathologic T stage, Pathologic N stage, Pathologic M stage, Histological type, Gender, CA19-9 level, Residual tumor, BMI, and Age (Table 1).

### Expression of SLC16A1 in Multi-Omics Data of Human Tumors

**Figure 1:**
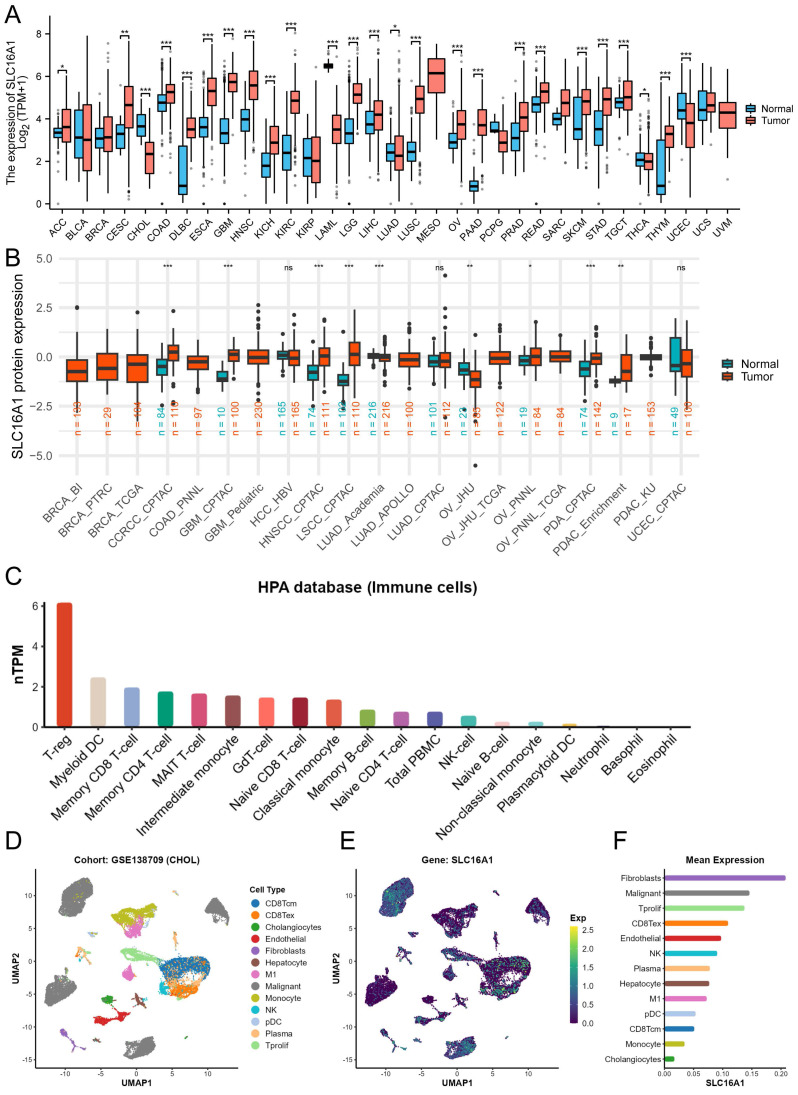
SLC16A1 Expression Analysis in Human Cancer. A: Comparative analysis of SLC16A1 mRNA expression in various cancers versus normal tissues, using TCGA and GTEx databases. B: Differential protein expression of SLC16A1 in human tumors versus normal tissues, as sourced from the CPTAC database. C: Expression levels of SLC16A1 across different immune cells, based on data from the HPA database. D-F: Single-cell analysis of SLC16A1 expression in the CCA tumor microenvironment, utilizing dataset GSE138709 from TISCH database.

To gain a foundational understanding of SLC16A1 expression in human tumors, we initially compared its mRNA expression levels in cancerous and corresponding normal tissues using the TCGA and GTEx databases. Our analysis revealed that SLC16A1 expression was significantly higher in various cancers, including ACC, CESE, CESC, COAD, DLBC, ESCA, GBM, HNSC, KIRC, KICH, LGG, TGCT, SKCM, LIHC, LUSC, OV, PAAD, READ, STAD, and THYM, compared to their respective normal tissues. Conversely, SLC16A1 showed significantly lower expression in CHOL, UCEC, LAML, and LUAD than in corresponding normal tissues (Figure 1A).

Furthermore, to clarify the protein expression levels of SLC16A1 in human tumors, we compared its expression in cancerous and corresponding normal tissues using data from the CPTAC database. The results indicated that SLC16A1's protein expression was higher in the cancerous tissues of ccRCC, GBM, HNSCC, LSCC, PDA, and PDAC compared to normal tissues. Interestingly, SLC16A1's protein expression in OV varied between cohorts: it was lower in tumor tissues than in normal tissues in the JHU cohort, whereas it was higher in the PNNL cohort (Figure 1B).

To explore SLC16A1 expression in different immune cells, we utilized data from the HPA database. The results showed that SLC16A1 had the highest expression in T-reg cells but was almost non-existent in Neutrophils, Basophils, and Eosinophils (Figure 1C). Lastly, using single-cell datasets (GSE138709) from TISCH, we studied the expression and distribution of SLC16A1 across different cell types in the tumor microenvironment of CCA at the single-cell level. It was observed that SLC16A1 was expressed in almost all constituent cell types of the CCA tumor microenvironment (Figure 1D-F). Notably, its expression was particularly high in malignant cells of CCA, second only to fibroblasts, yet it was lowest in normal bile duct cells (Figure 1E).

Additionally, the results of IHC revealed that SLC16A1 is also expressed at higher levels in an inflammatory background (cholangitis) compared to normal tissue. Furthermore, there is a significant increase in the expression of SLC16A1 in both the peritumoral tissues and the tumor tissues of cholangiocarcinoma, with expression observed in both the cytoplasm and cell membranes (Figure S1).

### Clinical Significance of SLC16A1 in CCA

**Figure 2:**
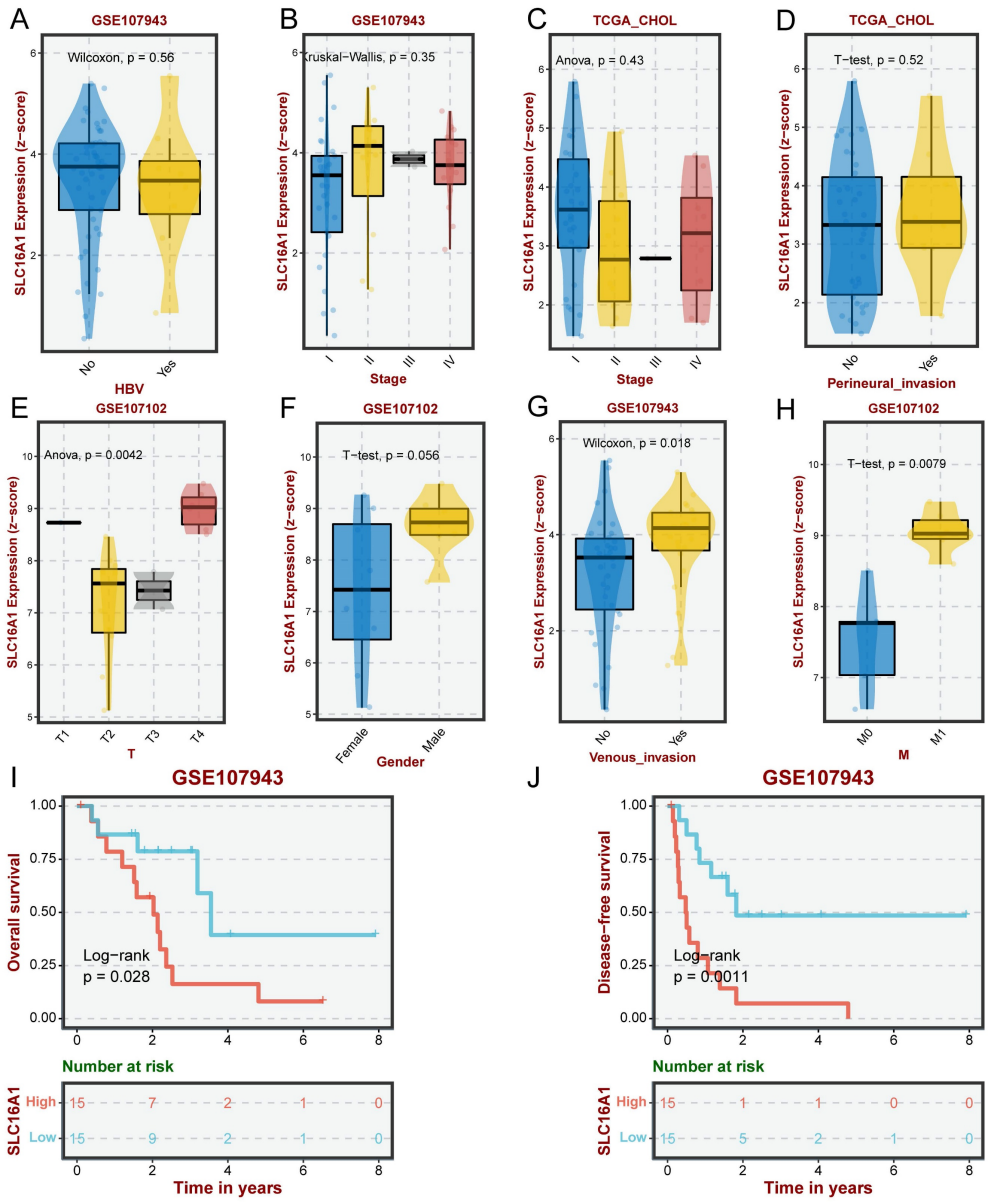
Impact of SLC16A1 Expression on CCA Clinicopathologic Feature. A-D: Analysis of SLC16A1 expression in CCA in relation to HBV infection status, pathological stage, and perineural invasion, showing no significant differences. E-H: Correlation of SLC16A1 expression with T stages and M stages in CCA, indicating higher expression at advanced stages and in cases with venous invasion. I-J: Kaplan-Meier survival analysis illustrating the relationship between SLC16A1 expression levels and the prognosis of CCA patients, with higher expression associated with poorer outcomes.

Previous research has demonstrated that SLC16A1, as an oncogene, exhibits varying levels of expression in many human tumors, with a particular focus on its significantly higher expression in the malignant cells of CCA compared to normal cells. This led us to investigate the clinical significance of SLC16A1 expression in CCA further. Our analysis indicated that the expression level of SLC16A1 did not show significant differences across various groups based on HBV infection status, pathological stage, or perineural invasion (Figure 2A-D). However, significant differences in SLC16A1 expression were observed across different T stages and M stages; specifically, higher stages correlated with higher SLC16A1 expression levels. Additionally, SLC16A1 expression was significantly elevated in cases with venous invasion compared to those without (Figure 2E-H).

Furthermore, Kaplan-Meier survival analysis revealed that the level of SLC16A1 expression was associated with the prognosis of CCA patients. Patients with high SLC16A1 expression had a poorer prognosis (Figure 2I-J). Overall, our results suggest that SLC16A1 may act as an oncogene, promoting tumor growth and invasion, thereby impacting the clinical outcomes of CCA patients.

### Correlation between SLC16A1 and Immune Infiltration Levels

**Figure 3:**
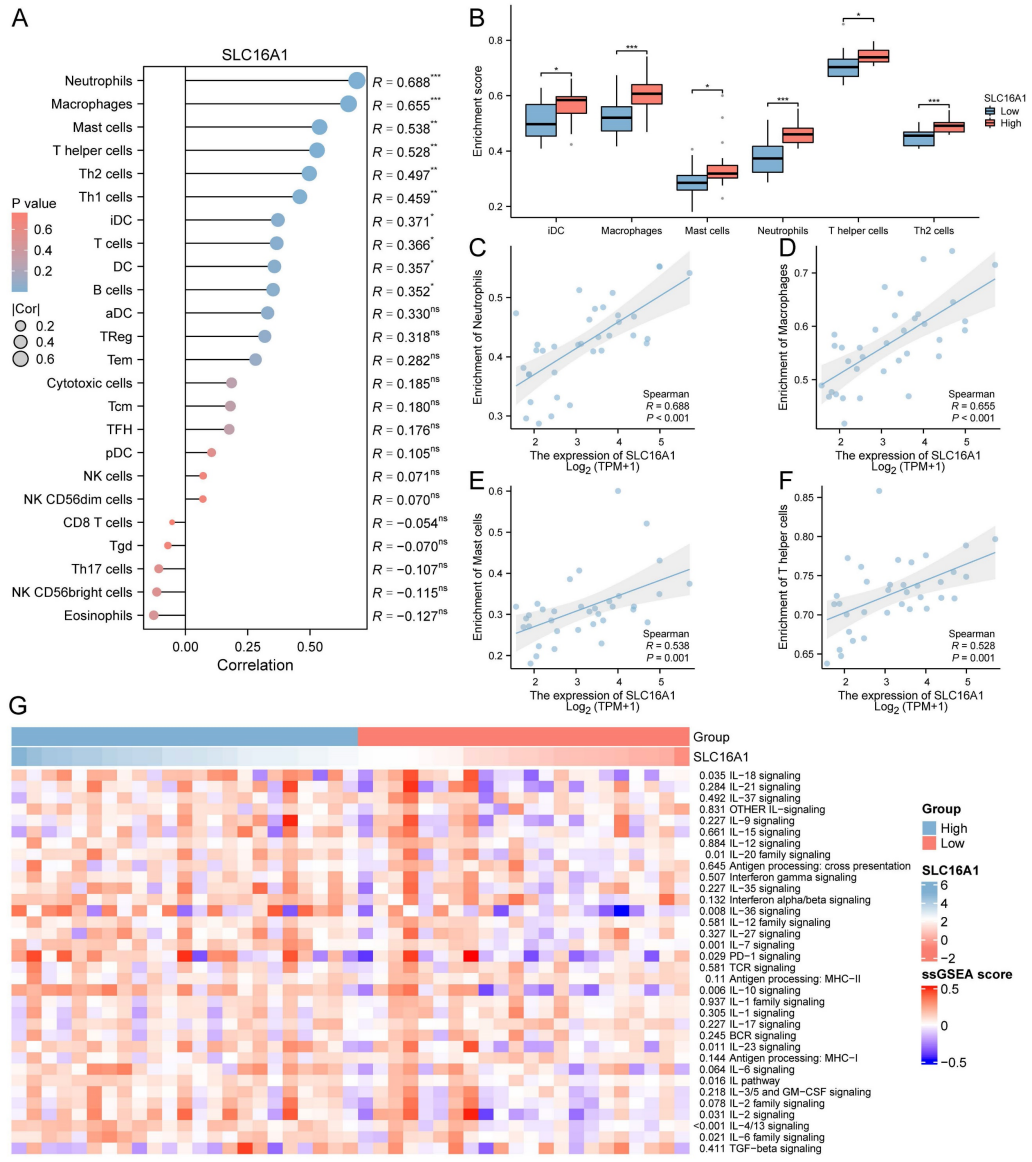
Correlation between SLC16A1 Expression and Immune Cell Infiltration in CCA. A: Correlation analysis between SLC16A1 expression and infiltration levels of various immune cells in CCA, using Spearman's method. B: Comparison of infiltration levels of Neutrophils, Macrophages, Mast cells, T helper cells, Th2 cells, and iDCs in high versus low SLC16A1 expression groups in CCA. C-F: Visualization of the correlation between SLC16A1 expression and the four most strongly associated immune cell types. G: Expression heatmap quantifying immune pathway activity differences between high and low SLC16A1 expression groups in CCA, based on the TCGA-CHOL dataset using ssGSEA.

Previous studies have indicated that SLC16A1 may play a role in the remodeling of the tumor immune microenvironment by regulating lactate. Based on this, we hypothesized that SLC16A1 might also be significant in the immune regulation of CCA patients. To explore this, we employed the ssGSEA method to analyze the infiltration of 24 types of immune cells in the tumor microenvironment of CCA patients. The correlation between SLC16A1 expression and the degree of infiltration of these immune cells was assessed using Spearman's method. Overall, the results indicated a significant positive correlation between the expression level of SLC16A1 and the infiltration levels of Neutrophils, Macrophages, Mast cells, T helper cells, Th2 cells, Th1 cells, iDC, T cells, B cells, and DCs (Figure 3A). Notably, the infiltration levels of Neutrophils, Macrophages, Mast cells, T helper cells, Th2 cells, and iDCs were significantly higher in the SLC16A1 high-expression group compared to the low-expression group (Figure 3B). We also visualized the correlation of the four cell types most strongly associated with SLC16A1 expression levels (Figure 3C-F). Additionally, we quantified the immune pathway activity in the TCGA-CHOL dataset using the ssGSEA method and constructed an expression heatmap. The analysis revealed significant differences in immune pathway activities between the high and low SLC16A1 expression groups in CCA patients (Figure 3G). Not only that, we also employed additional algorithms to evaluate the relationship between SLC16A1 expression and the level of immune cell infiltration across various cancers. The results indicated that the expression levels of SLC16A1 are correlated with the infiltration levels of multiple types of immune cells, as determined by different algorithms (Figure S2). These findings suggest that SLC16A1 may be involved in the regulation of the tumor immune microenvironment in CCA.

### Potential Mechanism of SLC16A1 in CCA

**Figure 4:**
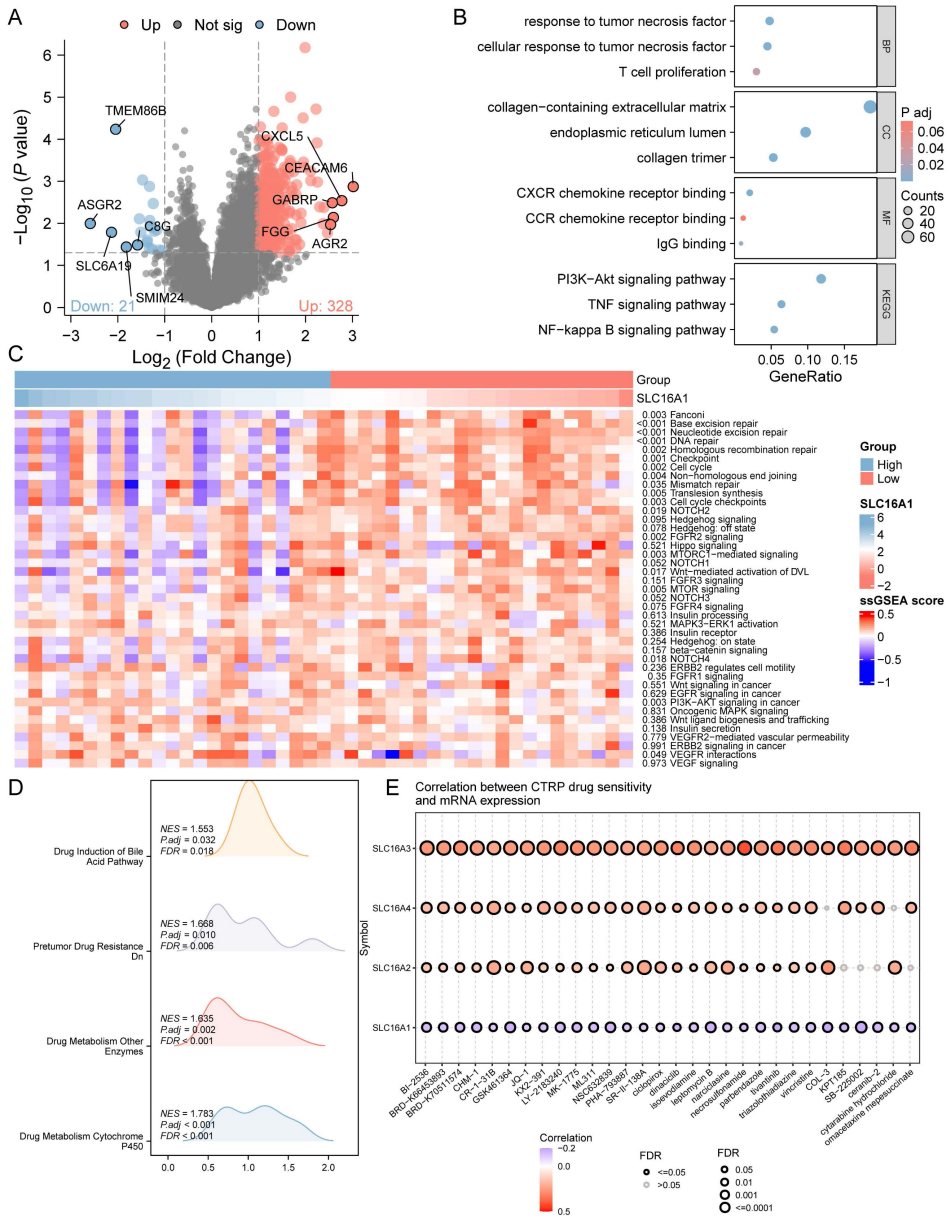
Functional Analysis of SLC16A1 in CCA. A: Volcano plot depicting the upregulated and downregulated genes between high and low SLC16A1 expression groups in CCA. B: Functional enrichment analysis showing significant involvement in immune regulation and cancer-related signaling pathways in CCA based on differential gene expression. C: Expression heatmap illustrating the activity differences in cancer-related pathways between high and low SLC16A1 expression groups in CCA, using the TCGA-CHOL dataset and ssGSEA. D: GSEA method analysis highlighting enriched pathways related to drug resistance in the high SLC16A1 expression group of CCA patients. E: Correlation between IC50 of small molecule compounds and expression of monocarboxylate transporters (SLC16A1, SLC16A2, SLC16A3, SLC16A4) in CCA, showing a negative correlation for SLC16A1 with top-ranked small molecules.

To delve deeper into the role of SLC16A1 in CCA, we analyzed the differential genes between high and low SLC16A1 expression groups in CCA patients. According to our set threshold, we identified 328 upregulated and 21 downregulated genes and further highlighted the top 5 upregulated and downregulated genes, as depicted in the volcano plot (Figure 4A). Subsequent functional enrichment analysis of these differential genes revealed significant enrichment in immune cell regulation pathways such as T cell proliferation, CXCR chemokine receptor binding, CCR chemokine receptor binding, and IgG binding (Figure 4B). These findings align with our earlier analysis, suggesting SLC16A1's role in immune regulation in CCA patients. Moreover, differential genes were also enriched in critical cancer-related signaling pathways, including the PI3K-Akt, TNF, and NF-kappa B signaling pathways (Figure 4B).

We further quantified the activity of cancer-related pathways in the TCGA-CHOL dataset using the ssGSEA method and constructed an expression heatmap. Comparative analysis revealed significant differences in cancer-related pathway activities between different groups (Figure 4C). These results underscore the potential involvement of SLC16A1 in the regulation of the tumor immune microenvironment in CCA and its potential as an oncogene.

Given past studies suggesting SLC16A1's role in drug resistance across various tumors, we employed the GSEA method to explore whether SLC16A1 influences drug resistance in CCA patients. The analysis indicated that pathways related to drug resistance, such as Drug Induction of Bile Acid Pathway, Pretumor Drug Resistance Dn, Drug Metabolism of Other Enzymes, and Drug Metabolism Cytochrome P450, were significantly enriched in the SLC16A1 high expression group (Figure 4D). Additionally, we analyzed the correlation between the IC50 of small molecule compounds and the expression of four monocarboxylate transporters (SLC16A1, SLC16A2, SLC16A3, SLC16A4) using the CTRP database. Drugs were ranked based on their correlation coefficients and FDR levels with these genes, and the top 30 drugs are illustrated in the figure. Interestingly, we observed that the expression levels of other monocarboxylate transporters in the SLC16 family (SLC16A2, SLC16A3, SLC16A4) were positively correlated with the IC50 of the top-ranked small molecules. However, the expression of SLC16A1 showed a negative correlation with the IC50 of these leading small molecules (Figure 4E).

### SLC16A1 Knockdown Leads to Reduced CCA Growth *In Vitro* and *In Vivo*

**Figure 5:**
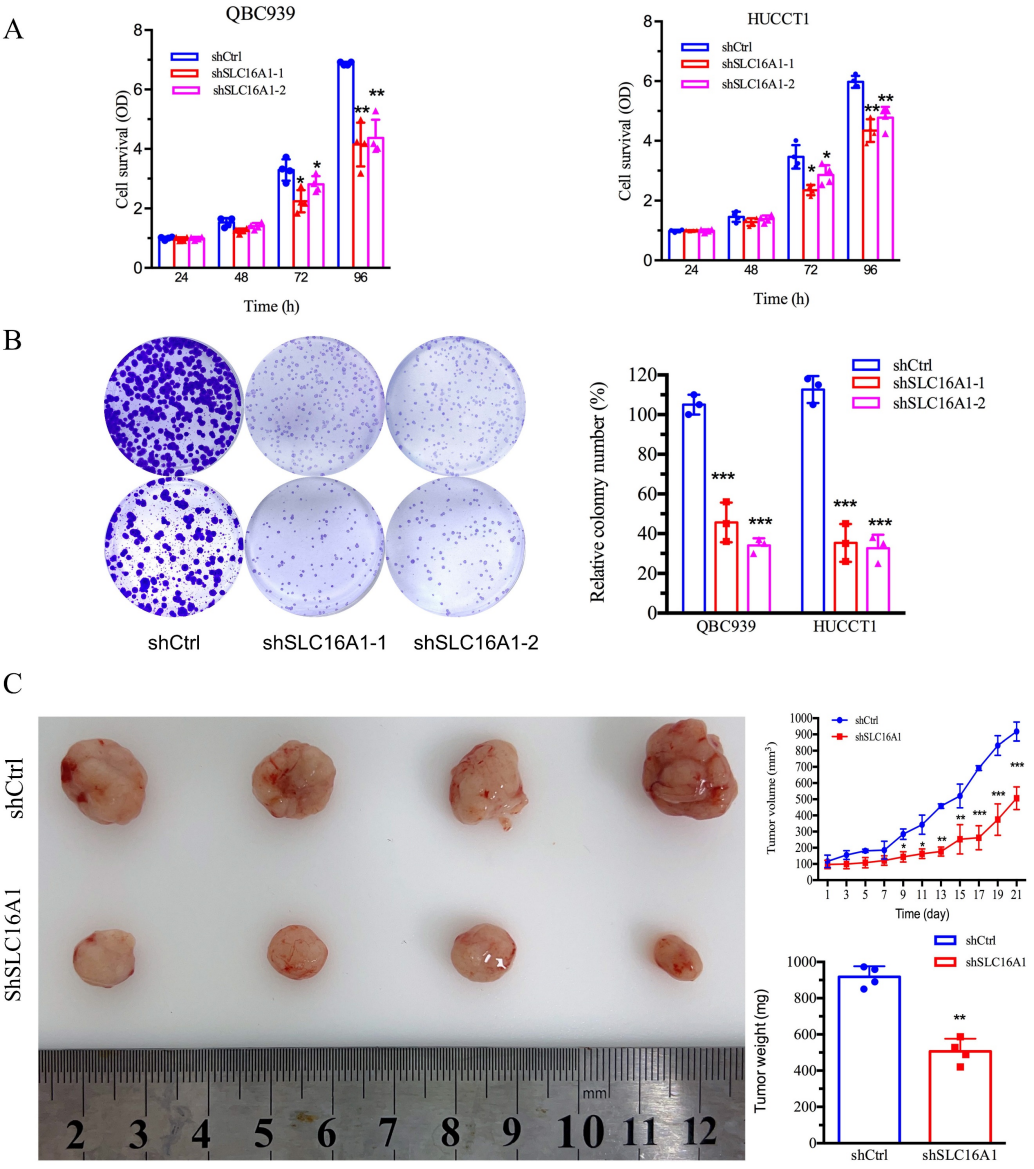
Inhibition of Tumor Cell Growth Following SLC16A1 Gene Knockdown In Vitro and In Vivo. A: Cell survival assays of QBC939 and HUCCT1 cell lines with SLC16A1 knockdown (shSLC16A1-1 and shSLC16A1-2) versus control (shCtrl), measured at 24, 48, 72, and 96 hours, showing reduced proliferation in SLC16A1-suppressed cells. B: Colony formation assay comparing the clonogenic capacity of QBC939 and HUCCT1 cells post-SLC16A1 knockdown versus control. Knockdown groups demonstrate a significant decrease in colony number, indicating growth inhibition. C: In vivo tumor growth assay depicting the effect of SLC16A1 knockdown on tumor size and weight in a xenograft model. The left panel shows representative tumors harvested from control and SLC16A1 knockdown groups; the right panel presents quantified tumor volumes over time and final tumor weights, highlighting marked growth suppression in the shSLC16A1 group.

Our prior bioinformatics analysis suggested a potential oncogenic role for SLC16A1 in CCA. To further investigate this, we conducted *in vitro* and *in vivo* studies to assess the impact of SLC16A1 on tumor growth. The results indicated that knocking down SLC16A1 significantly reduced the growth of CCA both *in vitro* and *in vivo*. This was primarily evidenced by the marked inhibition of the cell survival and number of CCA cell colonies *in vitro* (Figure 5A-B), as well as the reduction in size and quantity of subcutaneous tumors in nude mice (Figure 5C). In addition, results from IHC staining demonstrated that compared to the control group, SLC16A1 Knockdown significantly reduced the number of Ki-67 positive cells in mouse tumor tissue and it also had a slight impact on the expression of Caspase-3 (Figure S3). These findings robustly support the hypothesis that SLC16A1 acts as an oncogene in CCA, promoting tumor growth and proliferation.

### SLC16A1 Induces 5-FU Resistance in CCA Cells

**Figure 6:**
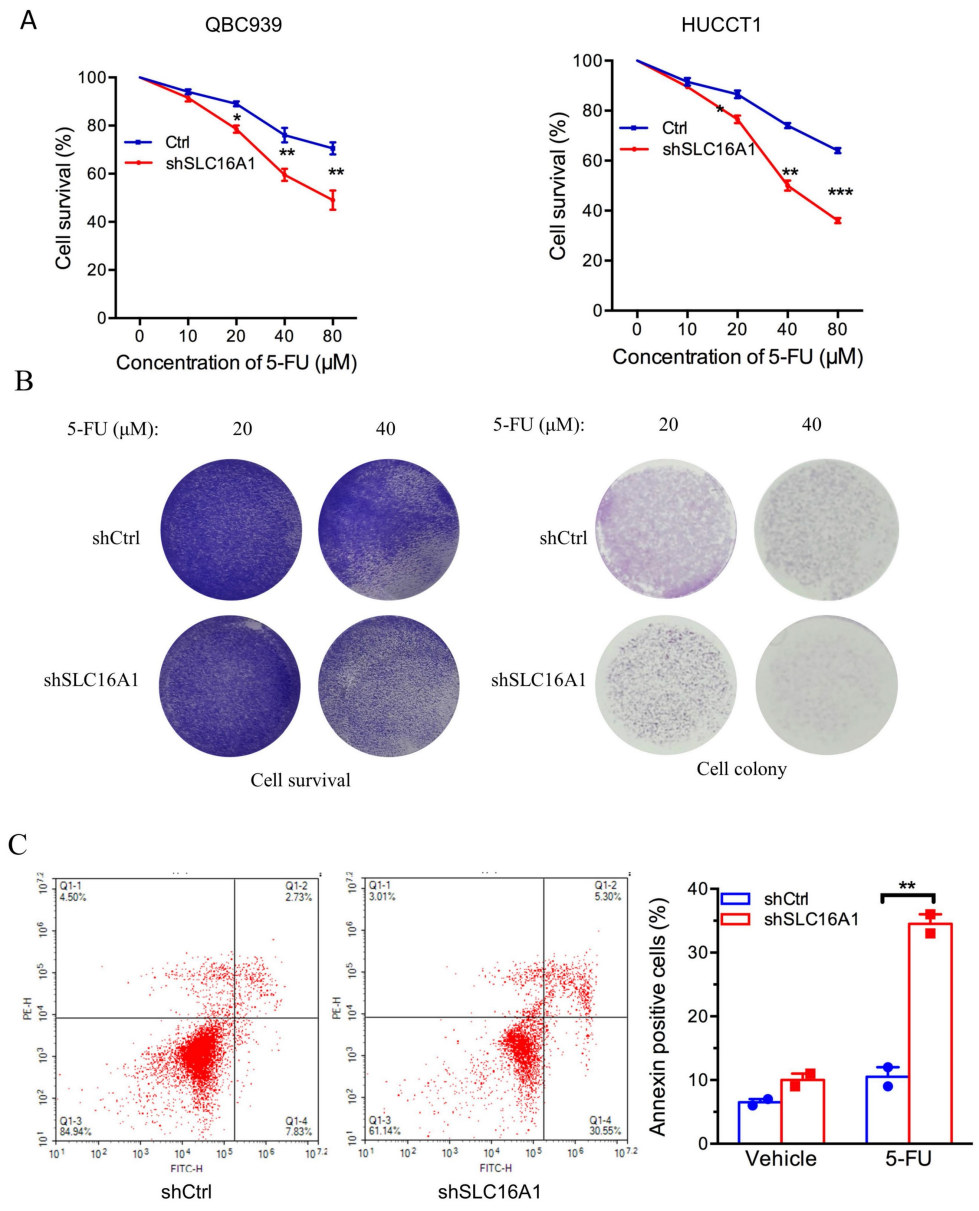
SLC16A1 Knockdown Sensitizes Tumor Cells to 5-FU Treatment In Vitro. A: Dose-response curves for QBC939 and HUCCT1 cell lines treated with varying concentrations of 5-FU, demonstrating decreased cell survival in shSLC16A1 cells compared to control (shCtrl). B: After treatment with 5-FU, 90% of the cells were used for the cell survival assay, while the remaining 10% were utilized for the clonogenic assay. This revealed a decreased survival rate and clonogenic capacity in shSLC16A1 cells compared to shCtrl. C: Flow cytometric analysis of apoptosis in QBC939 cells treated with vehicle or 5-FU, indicating a higher percentage of Annexin V-positive cells in the shSLC16A1 group compared to shCtrl, suggestive of enhanced apoptotic response upon SLC16A1 knockdown.

To further ascertain the influence of SLC16A1 on drug resistance, we investigated the impact of SLC16A1 expression on cell survival under different concentrations of 5-FU in QBC939 and HuCCT1 cells. CCK-8 assay results indicated that compared to the control group, the knockdown of SLC16A1 led to a gradual decrease in the survival rate of CCA cells with increasing concentrations of 5-FU (Figure 6A-B). Specifically, at a 5-FU concentration of 10 μM, the knockdown of SLC16A1 had no significant effect on the survival rate of both CCA cell lines compared to the control. However, at 5-FU concentrations of 20 μM, 40 μM, and 80 μM in QBC939 cells, the survival rate decreased by 20% in the control group, while it decreased by 45% in the SLC16A1 knockdown group. In HuCCT1 cells, the survival rate decreased by 28% in the control group and by 55% in the SLC16A1 knockdown group.

Furthermore, to more accurately evaluate the impact of SLC16A1 on 5-FU-induced apoptosis in CCA cells, we performed flow cytometry analysis using Annexin V-FITC and PI staining on QBC939 cells. The translocation of phosphatidylserine from the inner to the outer leaflet of the cell membrane is a hallmark of early apoptosis. Fluorescently labeled Annexin-V, which has a high affinity for PS, can identify early apoptotic cells. Additionally, as the cell membranes of early apoptotic and live cells remain intact, propidium iodide (PI) can only penetrate necrotic cells (mid-to-late apoptotic cells), thus differentiating between early and late apoptosis.

Following 24 hours of 5-FU treatment of QBC939 cells, we stained the cells with Annexin V-FITC and PI. Flow cytometry results showed that compared to the control group, the knockdown of SLC16A1 significantly increased the rate of early apoptotic cells induced by 5-FU treatment (from 7.83% to 30.55%) and reduced the survival rate (from 84.94% to 61.14%) (Figure 6C). In summary, knocking down SLC16A1 enhances the sensitivity of CCA cells to 5-FU treatment.

## Discussion

Previous research has already highlighted the critical role of SLC16A1 in the development and progression of various cancers. However, the precise function of SLC16A1 in CCA remained unclear. In the present study, through bioinformatics analysis, we began to unravel the potential signaling pathways and immune regulation mechanisms involving SLC16A1 in CCA. Further *in vitro* and *in vivo*, experimental results have demonstrated that the knockdown of SLC16A1 effectively inhibits the growth of CCA cells, affirming its significant role as an oncogene in this type of cancer. Importantly, the knockdown of SLC16A1 also increased the sensitivity of CCA cells to the commonly used chemotherapeutic agent 5-FU. Overall, this study not only reveals the key role of SLC16A1 in the development of CCA but also highlights its potential as a target for improving treatment outcomes and chemotherapy sensitivity in CCA. This research contributes substantially to our understanding of CCA and opens up new avenues for therapeutic strategies targeting SLC16A1.

Although some studies have investigated the expression of SLC16A1 in human cancers, the information available remains incomplete. Therefore, we initially utilized bioinformatics methods to analyze the mRNA and protein level expression of SLC16A1 in human cancers. We found that SLC16A1's mRNA expression levels were generally elevated across a variety of human cancers. However, at the protein expression level, significant differential expression of SLC16A1 was only observed in certain urological tumors 17, 18, such as renal clear cell carcinoma 19, 20, which is consistent with previous research findings. Interestingly, in our study, we observed divergent results in the protein expression levels of SLC16A1 across different ovarian cancer cohorts. We believe that this discrepancy may be attributed to tumor heterogeneity. Additionally, factors such as sample size and the staging and classification of tumors at the time of sample selection might also contribute to these findings. Therefore, further experimental validation using clinical samples to confirm the protein expression levels of SLC16A1 in CCA tissues is necessary.

Given the abnormal expression patterns of SLC16A1 observed in various human tumors, we further explored the clinical significance of SLC16A1's expression changes in CCA patients and its impact on the functionality of CCA cells. SLC16A1 has been identified as an oncogene in previous studies 21, 22. For instance, in renal clear cell carcinoma, targeting and blocking SLC16A1 can downregulate lactate flux, thereby inhibiting the proliferation and invasion capabilities of the cancer 23. In osteosarcoma, SLC16A1 suppression has been linked to anti-tumor potential related to the NF-κB pathway 9, with high SLC16A1 expression indicating a poor overall survival rate in patients 24. Additionally, studies have reported that SLC16A1, besides its function as a proton transporter, can also induce epithelial-mesenchymal transition in tumor cells by activating the HGF/C-MET pathway, thereby promoting tumor invasion and metastasis 25. However, due to the heterogeneity of tumors, the role of SLC16A1 in CCA is still not clear and requires further elucidation. In our study, bioinformatics analysis revealed that SLC16A1-related differential genes in CCA are associated with numerous oncogenic pathways. Through further *in vitro* and *in vivo* experimental validation, we confirmed the significant role of SLC16A1 as an oncogene in CCA. Considering that the mechanisms of action of SLC16A1 vary with tumor types in previous studies, future research needs to combine bioinformatics analysis to explore the mechanisms of SLC16A1 further. This will help us better understand its role in CCA and guide its clinical application.

In our previous studies, we discovered that SLC16A1 plays a crucial role in driving the progression of tumor cells. However, a major challenge in clinical treatment is the development of drug resistance in tumor cells as the cancer progresses. We noted that the function of SLC16A1 extends beyond simple transport; it also plays a vital role in maintaining cellular pH balance and is an essential component in various metabolic processes, including aerobic glycolysis 26, 27. Numerous studies have shown that modulating and altering metabolic pathways like aerobic glycolysis is crucial to enhancing the sensitivity of many cancer cells to chemotherapy drugs. Furthermore, past research has demonstrated SLC16A1's significant role in drug resistance in colon cancer and breast cancer 28, 29, indicating its potential involvement in tumor drug resistance. Therefore, we hypothesize that SLC16A1 might indirectly affect the sensitivity of tumor cells to anticancer drugs by regulating their metabolic pathways. Preliminary data analysis from databases revealed a correlation between SLC16A1 expression levels and chemotherapy drug sensitivity. However, unlike other members of the monocarboxylate transporter family, high expression of SLC16A1 is significantly negatively correlated with the IC50 values of various small molecule inhibitors, suggesting that high SLC16A1 expression may enhance the chemotherapy effect of certain drugs.

To bring our research closer to clinical application, we chose 5-FU for experimentation to verify the impact of SLC16A1 expression levels on tumor cell drug sensitivity. 5-FU, commonly used in the treatment of CCA, typically has limited effectiveness against this disease 30. Notably, our analysis shows that cells with knocked-down SLC16A1 are more sensitive to 5-FU, indicating that high SLC16A1 expression may increase the resistance of CCA cells to 5-FU. Although bioinformatics analysis suggests that SLC16A1 expression levels might be significantly negatively correlated with the IC50 levels of most drugs, this could be related to the physicochemical properties of small molecule compounds. However, based on these results, we believe that combining bioinformatics analysis with specific validation of currently developed clinical drugs can better help us understand the role of SLC16A1 in the clinical treatment of CCA.

Although our research data highlights the critical role of SLC16A1 in CCA, specifically revealing that abnormal expression of SLC16A1 is not only closely related to the malignancy of tumor cells but may also increase their chemoresistance, there are some limitations in our study: 1) The bioinformatics analysis methods and the number of samples used were limited; 2) There was an insufficient number of clinical samples; 3) The study did not delve deeply enough into the underlying signaling mechanisms of SLC16A1 in CCA. Further research addressing these issues will provide more insights into the role of SLC16A1 in the drug resistance mechanisms of CCA and its important role in tumor development and progression. Overall, our research provides new insights into the role of SLC16A1 in CCA cells, which may promote the development of new therapeutic strategies to overcome the drug resistance of CCA cells to existing cancer treatments. To our knowledge, this is the first study to describe the role of SLC16A1 in drug resistance in CCA, and targeting SLC16A1 with combination therapy or developing specific inhibitors may become an effective strategy to improve the treatment of CCA.

## Supplementary Material

Supplementary figures.

## Figures and Tables

**Table 1:** Characteristic of 35 CCA patients from the TCGA database

Characteristics	Overall
Pathologic T stage, n (%)	
T1&T2	30 (85.7%)
T3&T4	5 (14.3%)
Pathologic N stage, n (%)	
N0	25 (83.3%)
N1	5 (16.7%)
Pathologic M stage, n (%)	
M0	27 (84.4%)
M1	5 (15.6%)
Histological type, n (%)	
Distal	2 (5.7%)
Hilar/perihilar	4 (11.4%)
Intrahepatic	29 (82.9%)
Gender, n (%)	
Female	19 (54.3%)
Male	16 (45.7%)
CA19-9 level, n (%)	
Normal	13 (44.8%)
Abnormal	16 (55.2%)
Residual tumor, n (%)	
R0	27 (84.4%)
R1	5 (15.6%)
BMI, n (%)	
<= 25	10 (29.4%)
> 25	24 (70.6%)
Age, n (%)	
<= 65	17 (48.6%)
> 65	18 (51.4%)

## Abbreviations

ACC: adrenocortical carcinoma
BLCA: bladder urothelial carcinoma
BRCA: breast invasive carcinoma
CESC: cervical squamous cell carcinoma and endocervical adenocarcinoma
CHOL: cholangiocarcinoma
COAD: colon adenocarcinoma
DLBC: lymphoid neoplasm diffuse large B-cell lymphoma
ESCA: esophageal carcinoma
GBM: glioblastoma multiforme
KICH: kidney chromophobe
KIRC, ccRCC: kidney renal clear cell carcinoma
KIRP: kidney renal papillary cell carcinoma
LAML: acute myeloid leukemia
LGG: brain lower grade glioma
LIHC: liver hepatocellular carcinoma
LUAD: lung adenocarcinoma
LUSC: lung squamous cell carcinoma
MESO: mesothelioma
OV: ovarian serous cystadenocarcinoma
PAAD: pancreatic adenocarcinoma
PCPG: pheochromocytoma and paraganglioma
PRAD: prostate adenocarcinoma
READ: rectum adenocarcinoma
SARC: sarcoma
SKCM: skin cutaneous melanoma
STAD: stomach adenocarcinoma
TGCT: testicular germ cell tumors
THCA: thyroid carcinoma
THYM: thymoma
UCEC: uterine corpus endometrial carcinoma
UCS: uterine carcinosarcoma
UVM: uveal melanoma
