# Supplementary material for: Exploring SLC16A1 as an Oncogenic Regulator and Therapeutic Target in Cholangiocarcinoma
Source: J Cancer. 2024 May 20;15(12):3794–808. doi: 10.7150/jca.95258 (PMC11190756; doi:10.7150/jca.95258)
Supplement: Supplementary file 1 — Supplementary figures. [file jcav15p3794s1.pdf]

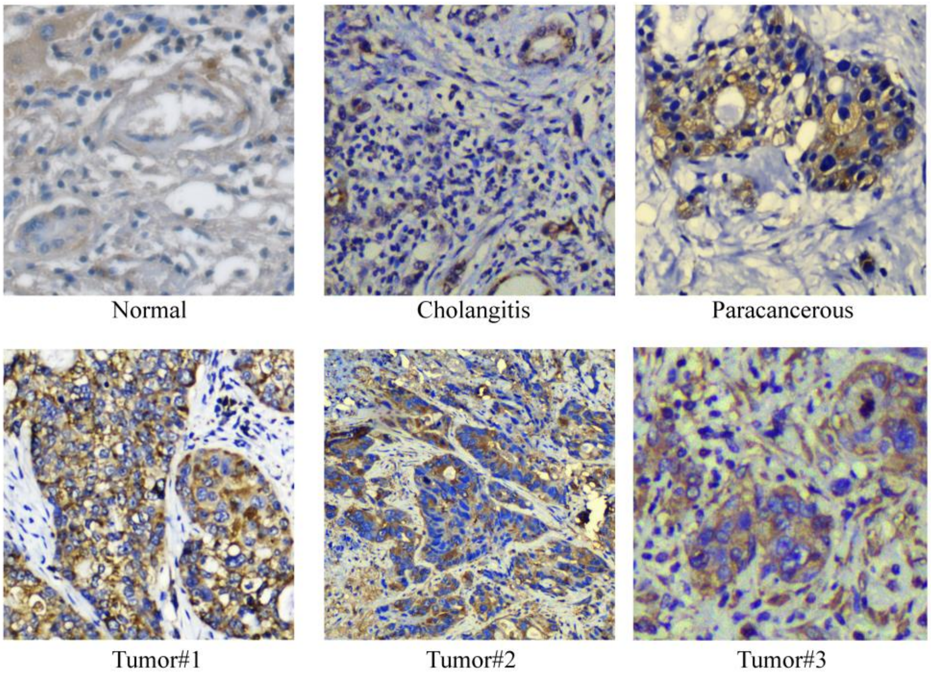

Figure S1. The IHC results indicate high expression of SLC16A1 in cholangiocarcinoma tissue (200X).

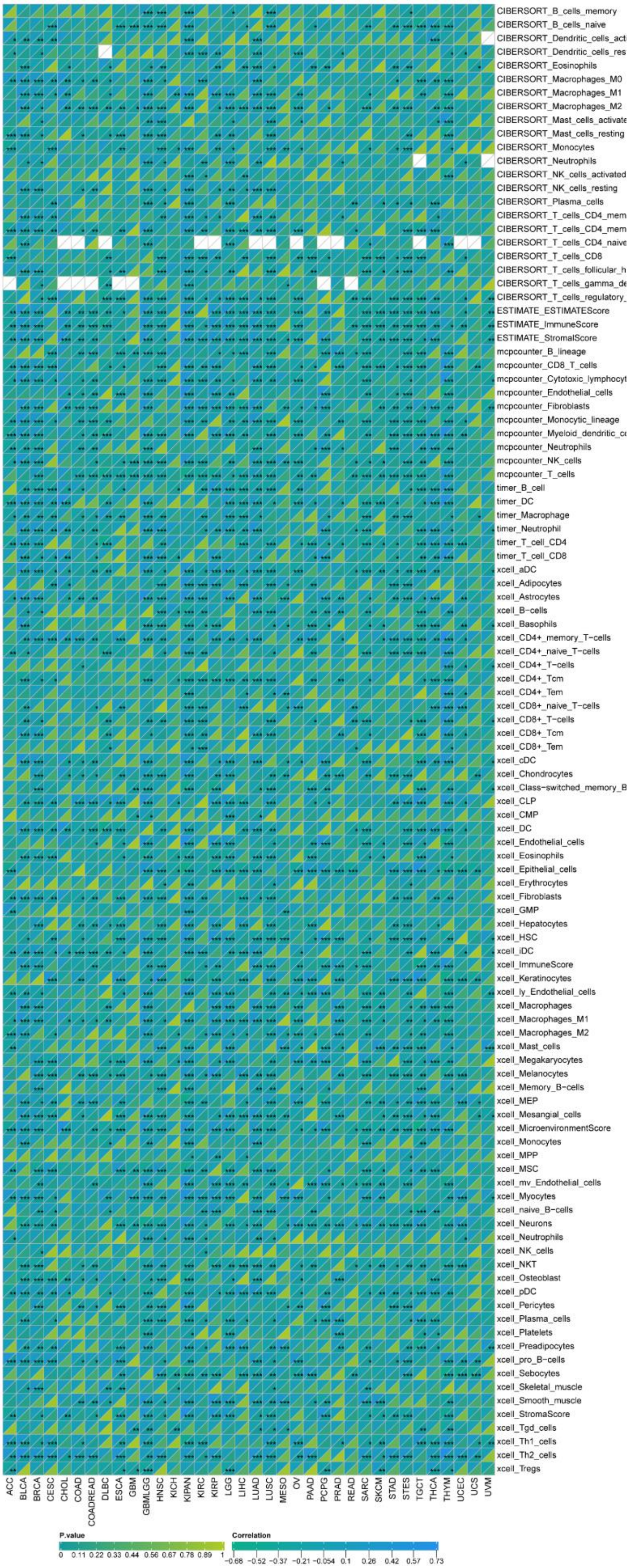

CIBERSORT_B_cells_memory
CIBERSORT_B_cells_naive
CIBERSORT_Dendritic_cells_acti
CIBERSORT_Dendritic_cells_res
CIBERSORT_Eosinophils
CIBERSORT_Macrophages_M0
CIBERSORT_Macrophages_M1
CIBERSORT_Macrophages_M2
CIBERSORT_Mast_cells_activate
CIBERSORT_Mast_cells_resting
CIBERSORT_Monocytes
CIBERSORT_Neutrophils
CIBERSORT_NK_cells_activated
CIBERSORT_NK_cells_resting
CIBERSORT_Plasma_cells
CIBERSORT_T_cells_CD4_mem
CIBERSORT_T_cells_CD4_mem
CIBERSORT_T_cells_CD4_naive
CIBERSORT_T_cells_CD8
CIBERSORT_T_cells_follicular_h
CIBERSORT_T_cells_gamma_de
CIBERSORT_T_cells_regulatory_
ESTIMATE_ESTIMATEScore
ESTIMATE_ImmuneScore
ESTIMATE_StromalScore
mcpcounter_B_lineage
mcpcounter_CD8_T_cells
mcpcounter_Cytotoxic_lymphocyt
mcpcounter_Endothelial_cells
mcpcounter_Fibroblasts
mcpcounter_Monocytic_lineage
mcpcounter_Myeloid_dendritic_ce
mcpcounter_Neutrophils
mcpcounter_NK_cells
mcpcounter_T_cells
timer_B_cell
timer_DC
timer_Macrophage
timer_Neutrophil
timer_T_cell_CD4
timer_T_cell_CD8
xcell_aDC
xcell_Adipocytes
xcell_Astrocytes
xcell_B-cells
xcell_Basophils
xcell_CD4+_memory_T-cells
xcell_CD4+_naive_T-cells
xcell_CD4+_T-cells
xcell_CD4+_Tcm
xcell_CD4+_Tem
xcell_CD8+_naive_T-cells
xcell_CD8+_T-cells
xcell_CD8+_Tcm
xcell_CD8+_Tem
xcell_cDC
xcell_Chondrocytes
xcell_Class-switched_memory_B
xcell_CLP
xcell_CMP
xcell_DC
xcell_Endothelial_cells
xcell_Eosinophils
xcell_Epithelial_cells
xcell_Erythrocytes
xcell_Fibroblasts
xcell_GMP
xcell_Hepatocytes
xcell_HSC
xcell_iDC
xcell_ImmuneScore
xcell_Keratinocytes
xcell_ly_Endothelial_cells
xcell_Macrophages
xcell_Macrophages_M1
xcell_Macrophages_M2
xcell_Mast_cells
xcell_Megakaryocytes
xcell_Melanocytes
xcell_Memory_B-cells
xcell_MEP
xcell_Mesangial_cells
xcell_MicroenvironmentScore
xcell_Monocytes
xcell_MPP
xcell_MSC
xcell_mv_Endothelial_cells
xcell_Myocytes
xcell_naive_B-cells
xcell_Neurons
xcell_Neutrophils
xcell_NK_cells
xcell_NKT
xcell_Osteoblast
xcell_pDC
xcell_Pericytes
xcell_Plasma_cells
xcell_Platelets
xcell_Preadipocytes
xcell_pro_B-cells
xcell_Sebocytes
xcell_Skeletal_muscle
xcell_Smooth_muscle
xcell_StromaScore
xcell_Tgd_cells
xcell_Th1_cells
xcell_Th2_cells
xcell_Tregs
ACC
BLCA
BRCA
CESC
CHOL
COAD
COADREAD
DLBC
ESCA
GBM
GBMLGG
HNSC
KICH
KIPAN
KIRC
KIRP
LGG
LIHC
LUAD
LUSC
MESO
OV
PAAD
PCPG
PRAD
READ
SARC
SKCM
STAD
STES
TGCT
THCA
THYM
UCEC
UCS
UVM
P.value
0
0.11
0.22
0.33
0.44
0.56
0.67
0.78
0.89
1
Correlation
−0.68
−0.52
−0.37
−0.21
−0.054
0.1
0.26
0.42
0.57
0.73

Figure S2. The association between the expression levels of SLC16A1 and the level of immune infiltration in pan-cancer.

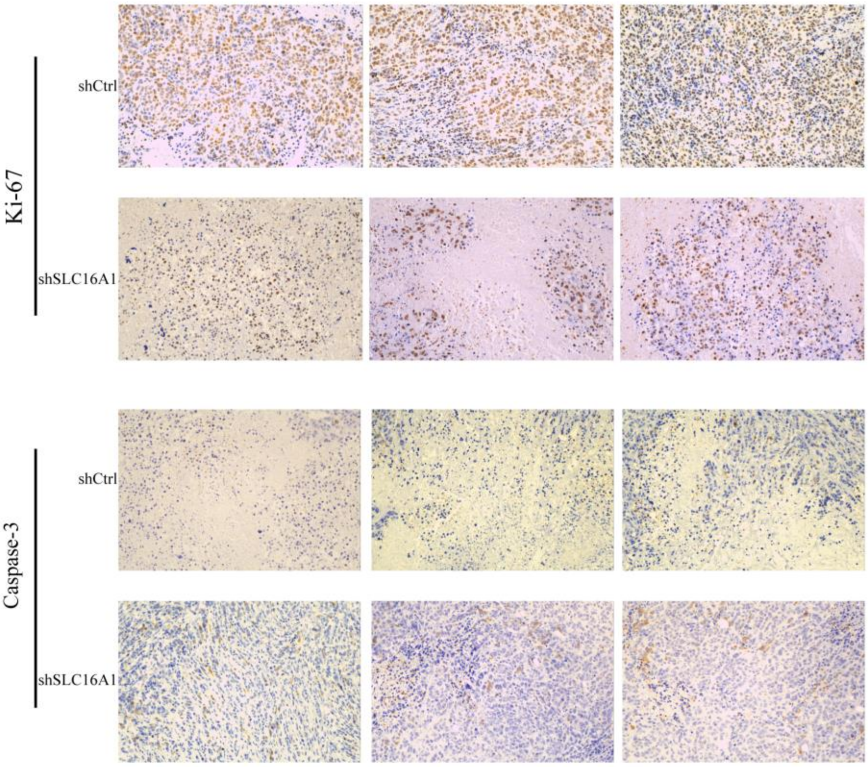

Figure S3. Representative figures of Ki-67 and Caspase-3 by IHC in sh-Ctrl and sh-SLC16A1 groups (100X).
